# Apatite-Forming Ability and Visible Light-Enhanced Antibacterial Activity of CuO-Supported TiO_2_ Formed on Titanium by Chemical and Thermal Treatments

**DOI:** 10.3390/jfb15050114

**Published:** 2024-04-24

**Authors:** Po-Cheng Sung, Taishi Yokoi, Masaya Shimabukuro, Takayuki Mokudai, Masakazu Kawashita

**Affiliations:** 1 Graduate School of Medical and Dental Sciences, Tokyo Medical and Dental University, Bunkyo-ku, Tokyo 113-8549, Japan; foster01506@gmail.com; 2 Institute of Biomaterials and Bioengineering, Tokyo Medical and Dental University, 2-3-10 Kanda-Surugadai, Chiyoda-ku, Tokyo 101-0062, Japan; yokoi.taishi.bcr@tmd.ac.jp (T.Y.); shimabukuro.bcr@tmd.ac.jp (M.S.); 3 Joining and Welding Research Institute, Osaka University, 11-1 Mihogaoka, Ibaraki, Osaka 567-0047, Japan; tmokudai.jwri@osaka-u.ac.jp; 4 Institute of Materials Research, Tohoku University, 2-1-1 Katahira, Aoba-ku, Sendai, Miyagi 980-8577, Japan

**Keywords:** titanium, CuO, TiO_2_, apatite, antibacterial activity, photocatalytic activity

## Abstract

Titanium with apatite-forming ability as well as antibacterial activity is useful as a component of antibacterial dental implants. When Ti was subjected to hydrogen peroxide (H_2_O_2_), copper acetate (Cu(OAc)_2_), and heat (H_2_O_2_-Cu(OAc)_2_-heat) treatments, a network structure of anatase and rutile titanium dioxide (TiO_2_) and fine copper oxide (CuO) particles was formed on the Ti surface. The resulting samples accumulated a dense and uniform apatite layer on the surface when incubated in simulated body fluid and showed enhanced antibacterial activity against *Escherichia coli* and *Staphylococcus aureus* under visible-light irradiation. Electron spin resonance spectra of H_2_O_2_-Cu(OAc)_2_-heat-treated samples showed that hydroxyl radicals (·OH) were generated from the samples, and the concentration of ·OH increased with increasing Cu concentration of the Cu(OAc)_2_ solution. The enhanced antibacterial activity of these samples under visible-light irradiation may be attributable to the generation of ·OH from samples. These results suggest that Ti implants obtained using H_2_O_2_-Cu(OAc)_2_-heat treatments and subjected to regular or on-demand visible-light irradiation may provide a decreased risk of peri-implantitis.

## 1. Introduction

With increases in the number of dental implants installed, peri-implant diseases have become more common. Such diseases can be broadly classified into peri-implant mucositis and peri-implantitis. Peri-implant mucositis, considered a precursor to peri-implantitis, is defined as a reversible inflammation that is localized to the surrounding mucosal tissue and does not involve bone resorption. On the other hand, peri-implantitis is defined as an irreversible inflammation that involves soft tissue inflammation and bone resorption around the implant [1]; prolonged peri-implantitis leads to implant failure. As assessed at greater than 5 years after dental implant placement, the prevalence of peri-implantitis is estimated to be 10% to 37% at the patient level and 7% to 27% at the implant level [2,3,4,5,6,7]. Classically, surgical treatment has been the main therapy for peri-implantitis; however, in recent years, chemotherapy using antibiotics and anti-inflammatory drugs has been employed to treat invasive and severe peri-implantitis [8,9]. However, surgical treatment is itself highly invasive, and there are concerns about the emergence of drug-resistant bacteria due to the use of antibiotics and anti-inflammatory drugs [10].

In recent years, there has been ongoing development of antimicrobial therapies using micromaterials and nanomaterials. Titanium-based metal organic frameworks (Ti-MOFs) have been investigated for use in therapeutic areas, including the treatment of bacterial infection, cancer, inflammation, and bone injury [11]; notably, MOF-loaded biohybrid magnetic microrobots have been shown to achieve good dispersion and targeted mobility with enhanced antibacterial efficiency [12]. Surface-modified nanomaterials also have been proposed to achieve passive or active targeted antibacterial effects [13,14]. Furthermore, materials that possess topographical features with high aspect ratios show bactericidal activity [15,16]. However, it remains unclear whether any of these materials are effective in treating peri-implantitis. The use of titanium dental implants with antibacterial activity as well as osteoconductivity has been shown to reduce the risk of peri-implantitis. To date, many surface treatments of Ti have been proposed to impart antibacterial activity as well as osteoconductivity to this metal [17,18,19,20,21,22,23,24,25]. Orthopedic implants coated with silver-containing hydroxyapatite [26] are employed clinically in Japan, but dental implants with both antibacterial activity and osteoconductivity are not yet in clinical use. If TiO_2_ with visible-light-responsive photocatalytic antibacterial activity were generated on the abutment of Ti dental implants, the risk of peri-implantitis might be reduced by regular or on-demand visible-light exposure in the dental clinic. One candidate for the visible light source is a light-emitting diode (LED) light used in teeth whitening, but this LED light has been shown to exhibit difficulty in penetrating the gingiva and reaching the abutment of the dental implants. Therefore, the visible light-responsive antibacterial activity of the dental implants would be expected to be enhanced when the abutment is not completely covered by periodontal tissue. In previous work, our laboratory has attempted to generate copper-doped titanium dioxide (TiO_2_) on Ti by chemical and thermal treatments [27,28]. This approach acknowledges that TiO_2_ generated by such strategies has been shown to bond to living bone [29] and that Cu-doped TiO_2_ demonstrates visible-light-induced photocatalytic antibacterial activity [30,31,32]. Some metals other than Cu, such as silver and zinc, also exhibit antibacterial effects, but the visible-light-induced antibacterial effects cannot be expected for Ag- or Zn-doped TiO_2_. In this context, we reported that Cu-doped TiO_2_ formed on Ti when Ti was subjected to (sequentially) immersion in a sodium hydroxide solution, immersion in a Cu-containing aqueous solution, and heating at 600 °C for 1 h. Apatite was observed to accumulate on the surface of the resulting Ti within 7 days of incubation in simulated body fluid (SBF) [27,28], and the resulting sample exhibited antibacterial activity against *Escherichia coli* under visible-light irradiation [28]. However, the apatite formed only on a part of the sample surface, indicating that the sample had limited apatite-forming ability. Furthermore, the antibacterial activity of the sample was not assessed against Gram-positive bacteria such as *Staphylococcus aureus*.

In the present study, we induced the formation of Cu-doped TiO_2_ on Ti by subjecting the Ti to (sequentially) immersion in a hydrogen peroxide solution, immersion in a copper acetate (Cu(OAc)_2_) solution, and thermal treatment. Previous work showed that a TiO_2_ layer is formed on Ti by sequential treatment with an aqueous NaOH solution, hot water, and heat [29,33], but surface treatments involving immersion in a H_2_O_2_ solution also are known to generate a TiO_2_ layer on Ti [34,35,36,37,38]. In that previous work, we found that the TiO_2_ layer (which forms on Ti following such treatment) accumulates apatite when the sample is incubated in SBF [34,36,37,38]; this apatite permits bonding to living bone [35]. Therefore, from the perspective of simplifying surface treatment, treatment with H_2_O_2_ solution was employed to induce the formation of a TiO_2_ layer on Ti in the present study. Furthermore, we used a Cu(OAc)_2_ solution for Cu doping into TiO_2_, a strategy that suppresses the undesired dissolution of the TiO_2_ layer that otherwise results from immersion in acidic Cu-containing solutions such as copper nitrate (Cu(NO_3_)_2_) [27]. We additionally evaluated the resulting samples for surface structure, apatite-forming ability, and Cu release. Furthermore, we investigated the generation by the samples of hydroxyl radicals (·OH), a reactive oxygen species (ROS), and assessed the antibacterial activity of samples against *S. aureus* as well as *E. coli*, both with and without visible-light irradiation.

## 2. Materials and Methods

### 2.1. Sample Preparation

Commercially pure Ti (99.5%) (Test Materials Co., Ltd., Tokyo, Japan) was employed in this study. The Ti was provided in the form of 8-mm diameter cylindrical rods, which subsequently were sliced into 1.0-mm thick sections. Following slicing, the surfaces of the disks were subjected to mechanical polishing using a diamond pad (No. 400; Maruto Instrument Co., Ltd., Tokyo, Japan). The polished Ti disks were washed ultrasonically, for 10 min per cycle, once with acetone (99%; Nacalai Tesque, Inc., Kyoto, Japan) and then twice with ultrapure water. The washed Ti disks were then dried at room temperature and atmospheric pressure. Each dried Ti disk was immersed in 10 mL of a 30 vol% H_2_O_2_ aqueous solution (FUJIFILM Wako Pure Chemical Corp., Osaka, Japan) in an ECK-50ML-R polypropylene tube (AS-ONE Corp., Osaka, Japan), and the tube then was heated at 80 °C for 1 h using a BW101 water bath (Yamato Scientific Co., Ltd., Tokyo, Japan). The samples were removed from the H_2_O_2_ aqueous solution and immersed in Cu(OAc)_2_ aqueous solutions at various concentrations (1, 10, or 100 mM) in a polypropylene tube at 80 °C for 24 h using the water bath. After the Cu(OAc)_2_ treatment, the disk was removed from the solution, washed with ultrapure water, and then dried at room temperature and atmospheric pressure. Each Ti disk that had been treated sequentially with the H_2_O_2_ and Cu(OAc)_2_ solutions was then heat-treated at 600 °C for 1 h using an FO101 electric furnace (Yamato Scientific Co., Ltd.). Surface treatments and the sample designations are listed in Table 1.

### 2.2. Surface Analysis of Samples

Many of the experimental protocols and conditions described in Section 2.2, Section 2.3, Section 2.4, Section 2.5 and Section 2.6 are essentially identical to those used in previous studies [28,39]. The surface morphology of each sample was observed using a JSM-7900F field emission scanning electron microscope (FE-SEM; JEOL, Tokyo, Japan). A mapping image of the sample was obtained using a JED-2300 energy dispersive X-ray spectrometer (JEOL) attached to the FE-SEM. The crystalline phase of the sample surface layer was identified using a MiniFlex600 X-ray diffractometer (XRD; Rigaku Corporation, Tokyo, Japan) with Cu Kα radiation.

The composition of the surface layer was evaluated using a JPS-9010MC X-ray photoelectron spectroscopy (XPS) instrument (JEOL). Monochromatic Mg Kα radiation (1253.6 eV) at 10 kV and 10 mA was used as the X-ray source. The C 1s photoelectron peak at 285.0 eV was used as a reference to calibrate the binding energy. The XPS peak was analyzed using CasaXPS (version 2.3.24; Casa Software, Ltd., Devon, UK). The chemical state of Cu on the samples was determined using a modified Auger parameter, which was calculated as described previously [22]. We conducted background subtraction using the CasaXPS software, following Shirley’s method [40] to calculate the integrated intensity of peaks. The surface composition of the samples was calculated using CasaXPS while employing the following relative sensitivity factors: C 1s (1.00), O 1s (2.93), P 2p (1.192), Ca 2p (5.07), Ti 2p (7.81), and Cu 2p_3/2_ (16.73). The proportions of (Cu^0^ + Cu^+^) and Cu^2+^ at the sample surface were calculated using the following equations [41]:

% (Cu^0^ + Cu+) = (*A* − (*A*1*_s_*/*B_s_*) × *B*)/(*A* + *B*) × 100 (1)

% (Cu^2+^) = *B* (1 + (*A*1*_s_*/*B_s_*))/(*A* + *B*) × 100 (2)

where *A* is the total area of the Cu 2p_3/2_ peak, *B* is the area of the shake-up peak, and *A*1*_s_*/*B_s_* is the ratio of the Cu 2p_3/2_ peak/shake-up peak areas. In the present study, the *A*1*_s_*/*B_s_* ratio was fixed at the value of 1.89 expected for CuO.

### 2.3. Evaluation of In Vitro Apatite-Forming Ability of Sample

The apatite-forming ability of the samples was evaluated using an SBF [42,43] that contained ions at concentrations (Na^+^, 142.0 mM; K^+^, 5.0 mM; Ca^2+^, 2.5 mM; Mg^2+^, 1.5 mM; Cl^−^, 147.8 mM; HCO_3_^−^, 4.2 mM; HPO_4_^2−^: 1.0 mM; SO_4_^2−^, 0.5 mM) nearly identical to those found in human blood plasma. We prepared the SBF according to the ISO 23317:2014 [43] protocol. All chemicals used for SBF formulation were purchased from Nacalai Tesque, Inc. (Kyoto, Japan). A volume of 30 mL of the prepared SBF was dispensed into an ECK-50ML-R centrifuge tube. The samples were immersed in the SBF at 36.5 °C for 7 days. The samples were then removed from the SBF, gently rinsed with ultrapure water, and dried at approximately 25 °C and atmospheric pressure. The lower surface of each sample was observed using a VE8800 scanning electron microscope (SEM; Keyence Corp., Osaka, Japan) and XRD.

### 2.4. Measurement of Cu Ion-Release Behavior of Sample

**Figure 1:**
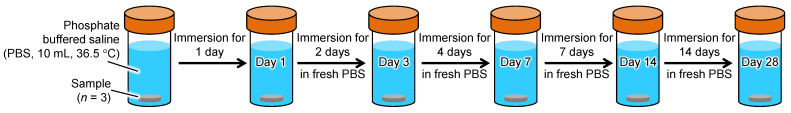
Schematic diagram of Cu ion-release assay. PBS, phosphate-buffered saline.

To investigate the Cu ion-release behavior of each sample, an aliquot (10 mL) of phosphate-buffered saline (PBS; Catalog 166-23555; FUJIFILM Wako Pure Chemical Corp., Osaka, Japan) was dispensed into an ECK-50ML-R centrifuge tube. The sample (*n* = 3) was immersed in PBS and the tube was maintained at 36.5 °C. The PBS was refreshed at appropriate periods. The amounts of Cu ions (per time point and cumulative) released from the samples were calculated at 1, 3, 7, 14, and 28 days based on the Cu concentrations in the PBS, which were measured using an ICP-7000 inductively coupled plasma atomic emission spectroscopy (ICP-AES) instrument (Shimadzu Corp., Kyoto, Japan). Figure 1 shows a schematic diagram of the Cu ion-release assay.

### 2.5. Evaluation of Antimicrobial Activity of Sample

The antibacterial activity of each sample was evaluated using strains of *S. aureus* (NBRC1221355; obtained from the NITE Biological Resource Center, Chiba, Japan) and *E. coli* (NBRC39725; also obtained from the NITE Biological Resource Center) according to the method described in JIS R 1752:2020 [44]. This experiment was approved by the Pathogenic Organisms Safety Management Committee of the Tokyo Medical and Dental University (Approval No. M22022-002; Approval date: 25 April 2022). *S. aureus* and *E. coli* were cultivated in soybean-casein digest (SCD) broth (Nissui Pharmaceutical Co., Ltd., Tokyo, Japan) or Luria–Bertani (LB) broth (MP Biomedicals, CA, USA), respectively, at 37 °C for 24 h. The resulting *S. aureus* cells were pelleted and resuspended in PBS at a density of 4.3 × 10^7^ to 1.0 × 10^8^ colony-forming units (CFU)/mL. Similarly, the resulting *E. coli* cells were pelleted and resuspended in PBS at a density of 3.2 × 10^7^ to 1.0 × 10^8^ CFU/mL. An antibacterial activity test was performed in quadruplicate (*n* = 4) for each sample, as described below.

Briefly, the sample (a Ti disc) was placed on a Falcon^®^ bacteriological petri dish (Catalog No. 351029; Corning, Inc., Corning, NY, USA) with the sample surface facing upward; an aliquot (10 μL) of the test bacterial suspension then was dispensed onto the sample. Subsequently, the sample surface was covered with plastic film (Catalog No. JPG533090; A.R. Medicom Inc. (Asia), Ltd., Kobe, Japan) to ensure close contact. The petri dish then was placed in a GB-2.0L gas barrier box (AS ONE Corp., Osaka, Japan). To reduce the effects on the bacteria of increasing temperature and drying during visible-light irradiation, pure water was dispensed into the bottom of the gas barrier box to maintain a relative humidity exceeding 90%. A 460-nm LED lamp (Model SPA-10SW; Hayashi Clock Industry Co., Ltd., Tokyo, Japan) was used as the light source. The distance from the lower part of the lens to the sample surface was 10 cm; the irradiance and the irradiation period were 250 W/m^2^ and 30 min, respectively. These irradiation parameters were defined based on the distance that might be expected between a visible-light source and the abutment of dental implants during dental treatment in the clinic. Following the visible-light irradiation, bacteria adhering to the sample and plastic film were recovered by washing with 1 mL of PBS. The resulting test bacterial suspension was subjected to 10-fold serial dilution in PBS, and aliquots (100 μL) of the 10-fold and 100-fold dilutions were plated on SCD agar (*S. aureus*) or LB agar (*E. coli*). After incubation at 37 °C for 24 h, the number of bacterial colonies formed on the plate surface was counted. The number of viable bacteria was calculated using the following formula:

*N* = *Z* × *E* × 10 (3)

where *N*, *Z*, and *E* are the viable bacterial count, arithmetic mean number of colonies, and dilution factor, respectively.

### 2.6. Measurement of ROS Generated by Samples

It is difficult to directly measure ROS and free radicals at room temperature (approximately 25 °C). Instead, we employed electron spin resonance (ESR) using a spin-trapping method to measure the ROS generated from samples irradiated with visible light. ESR was conducted using a JES-FA-100 instrument (JEOL, Ltd.). The spin-trapping agent consisted of 5,5-dimethyl-1-pyrroline-*N*-oxide (DMPO; Labotech Co., Tokyo, Japan). The ESR measurement was conducted under the following conditions: microwave power, 4.0 mW; microwave frequency, 9428.954 MHz; magnetic width, 0.1 mT; field sweep width, ±5 mT; field modulation frequency, 100 kHz; modulation width, 0.1 mT; time constant, 0.03 s; and sweep time, 0.1 min. Each sample was placed in a well of a 24-well plate, and 300 mM DMPO was dispensed at 500 μL/well. The samples immersed in the DMPO solution were irradiated with visible light, using the same 460-nm LED lamp for 30 min under the same conditions as those in the antibacterial property test. Subsequently, 200 μL of the DMPO solution (in which the sample had been immersed) was removed from each well and assessed for ROS using the ESR spectrometer. Hydroxyl radicals were quantified using 4-hydroxy-2,2,6,6-tetramethylpiperidine-1-oxyl (TEMPOL; Sigma-Aldrich, St. Louis, MO, USA) as the standard. The areas of DMPO-OH spectra were compared with that of a 2 μM TEMPOL standard measured under identical settings to estimate the concentration of the DMPO-OH adduct.

### 2.7. Statistical Analysis

Statistical analyses were performed using Microsoft Excel version 2209 (Microsoft, Redmond, WA, USA). One-way analysis of variance followed by multiple-hypothesis tests (Tukey’s honestly significant difference tests) was performed for comparisons between more than two groups. *p* < 0.01 was considered statistically significant.

## 3. Results

**Figure 2:**
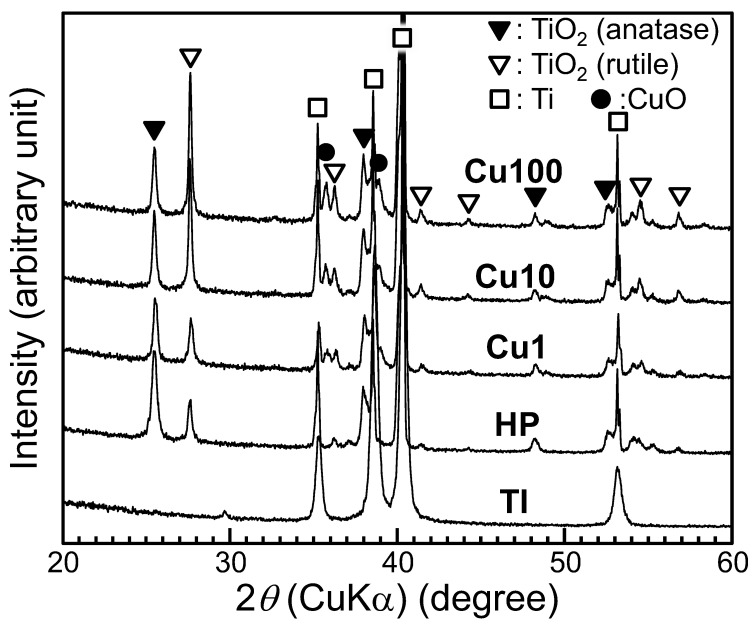
XRD patterns of samples. XRD: X-ray diffraction, TI: sample with no treatment, HP: sample with H_2_O_2_ treatment, Cu1: sample with H_2_O_2_, 1 mM Cu(OAc)_2_, and heat treatment, Cu10: sample with H_2_O_2_, 10 mM Cu(OAc)_2_, and heat treatment, Cu100: sample with H_2_O_2_, 100 mM Cu(OAc)_2_, and heat treatment.

XRD peaks attributable to anatase- and rutile-type TiO_2_ were observed for all samples other than Sample TI; peaks attributable to copper oxide (CuO) were observed for samples Cu1, Cu10, and Cu100 (Figure 2). As the Cu(OAc)_2_ concentration increased, the intensity ratio of the XRD peak of rutile-type TiO_2_ around 27.7° in 2*θ* to that of anatase-type TiO_2_ around 25.5° in 2*θ* also increased.

**Figure 3:**
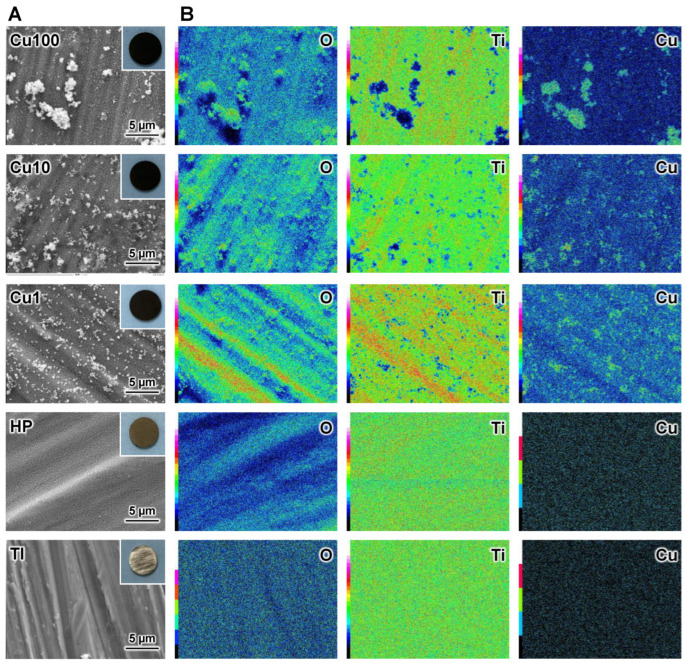
FE-SEM micrographs with insets showing macroscopic appearance (**A**), and EDS elemental mapping images (**B**) of samples. FE-SEM: field emission scanning electron microscope, EDS: energy dispersive X-ray spectrometer, TI: sample with no treatment, HP: sample with H_2_O_2_ treatment, Cu1: sample with H_2_O_2_, 1 mM Cu(OAc)_2_, and heat treatment, Cu10: sample with H_2_O_2_, 10 mM Cu(OAc)_2_, and heat treatment, Cu100: sample with H_2_O_2_, 100 mM Cu(OAc)_2_, and heat treatment. The color bar represents the intensity of EDS signal (black: low intensity, red or pink: high intensity).

FE-SEM micrographs (Figure 3A) showed that a fine network structure formed on the surface of the HP, Cu1, Cu10, and Cu100 samples and that fine particles formed on the Cu1, Cu10, and Cu100 samples. At the macroscopic level, the HP sample exhibited a brown color, while the Cu1, Cu10, and Cu100 samples exhibited a black color (insets in Figure 3A). The EDS mapping images of the samples (Figure 3B) indicated that the fine particles that formed on the Cu1, Cu10, and Cu100 samples were composed primarily of Cu and O.

**Figure 4:**
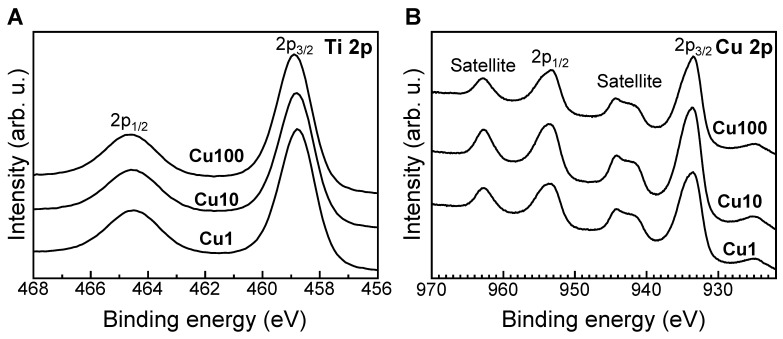
XPS narrow spectra in the Ti 2p (**A**) and Cu 2p (**B**) energy regions for the Cu1, Cu10, and Cu100 samples. XPS: X-ray photoelectron spectroscopy, arb. u.: arbitrary unit, Cu1: sample with H_2_O_2_, 1 mM Cu(OAc)_2_, and heat treatment, Cu10: sample with H_2_O_2_, 10 mM Cu(OAc)_2_, and heat treatment, Cu100: sample with H_2_O_2_, 100 mM Cu(OAc)_2_, and heat treatment.

In the XPS spectra of the Cu1, Cu10, and Cu100 samples, XPS peaks located in the C 1s, Ti 2p, O 1s, and Cu 2p energy regions were detected on the surfaces of the samples. The binding energy of the Ti 2p_3/2_ peak for the Cu1, Cu10, and Cu100 samples was approximately 458.8 eV (Figure 4A), indicating that Ti existed primarily as TiO_2_ in these samples [45,46]. The binding energies of the Cu 2p_3/2_ peaks for the Cu1, Cu10, and Cu100 samples were located at approximately 933.5, 933.7, and 933.7 eV, respectively (Figure 4B); the modified Auger parameters for the Cu1, Cu10, and Cu100 samples were measured at 1850.6, 1850.9, and 1850.7 eV, respectively. These results indicated that the Cu in these samples existed primarily as Cu^2+^ [22,41]. The concentrations of Cu on the surfaces of the Cu1, Cu10, and Cu100 samples were 6.8, 15.8, and 12.9 atomic%, respectively. For all three of these samples, the proportions of (Cu^0^ + Cu^+^) and Cu^2+^ ranged from 19.2 to 26.5% and 73.5–80.8%, respectively (Table 2).

**Figure 5:**
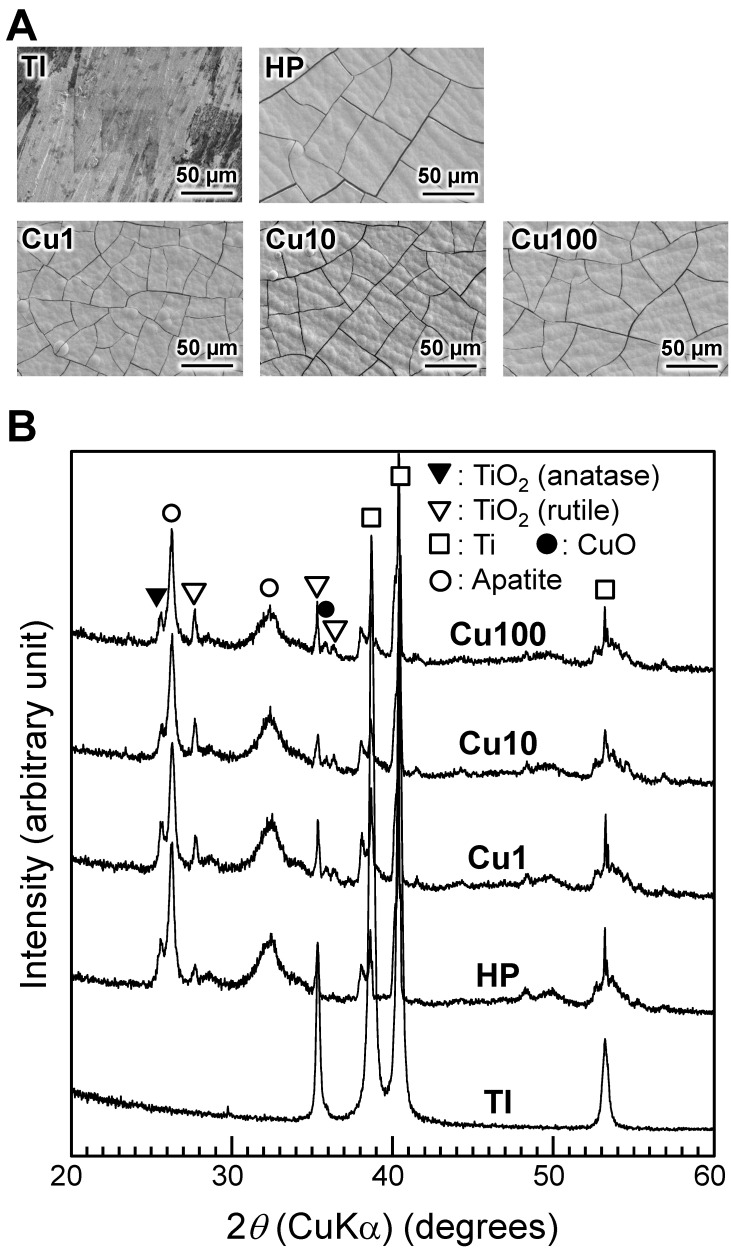
SEM photographs (**A**) and XRD patterns (**B**) of samples after 7 days of incubation in SBF. SEM: scanning electron microscope, TI: sample with no treatment, HP: sample with H_2_O_2_ treatment, Cu1: sample with H_2_O_2_, 1 mM Cu(OAc)_2_, and heat treatment, Cu10: sample with H_2_O_2_, 10 mM Cu(OAc)_2_, and heat treatment, Cu100: sample with H_2_O_2_, 100 mM Cu(OAc)_2_, and heat treatment, SBF: simulated body fluid.

SEM photographs and XRD patterns of samples following 7 days of incubation in SBF are shown in Figure 5. The HP, Cu1, Cu10, and Cu100 samples accumulated dense and uniform layers after a week of immersion in SBF, and XRD peaks attributable to apatite were observed for these samples.

**Figure 6:**
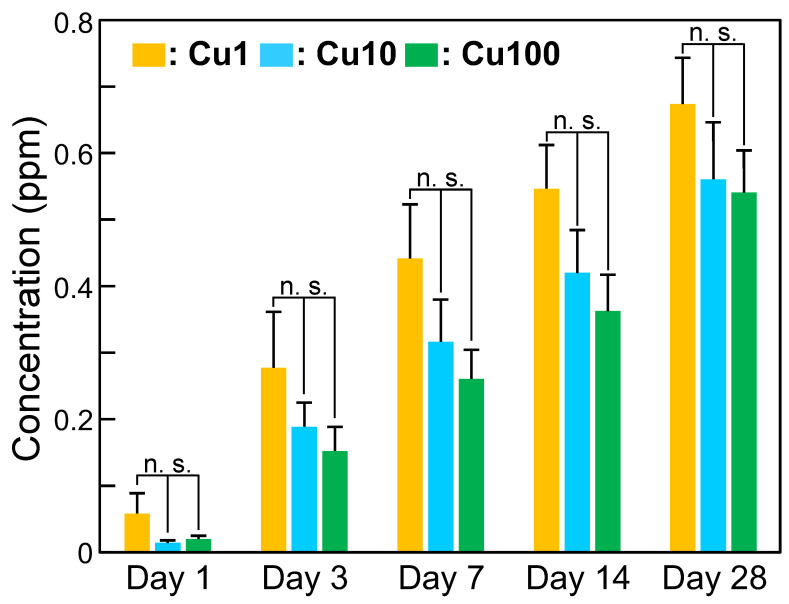
Concentration of Cu eluted from samples into PBS when immersed in PBS for periods of up to 28 days. Values are presented as arithmetic mean ± SD (*n* = 3). n.s. *p* ≥ 0.01 (one-way analysis of variance followed by multiple-hypothesis tests (Tukey’s honestly significant difference tests)). Cu1: sample with H_2_O_2_, 1 mM Cu(OAc)_2_, and heat treatment, Cu10: sample with H_2_O_2_, 10 mM Cu(OAc)_2_, and heat treatment, Cu100: sample with H_2_O_2_, 100 mM Cu(OAc)_2_, and heat treatment, PBS: phosphate-buffered saline, n.s.: not significant.

When samples were immersed in PBS for periods of up to 28 days, the concentration of Cu eluted from the samples increased gradually (Figure 6). However, no statistically significant differences were observed in the eluted Cu ion concentration when comparing the samples at a given immersion time point.

**Figure 7:**
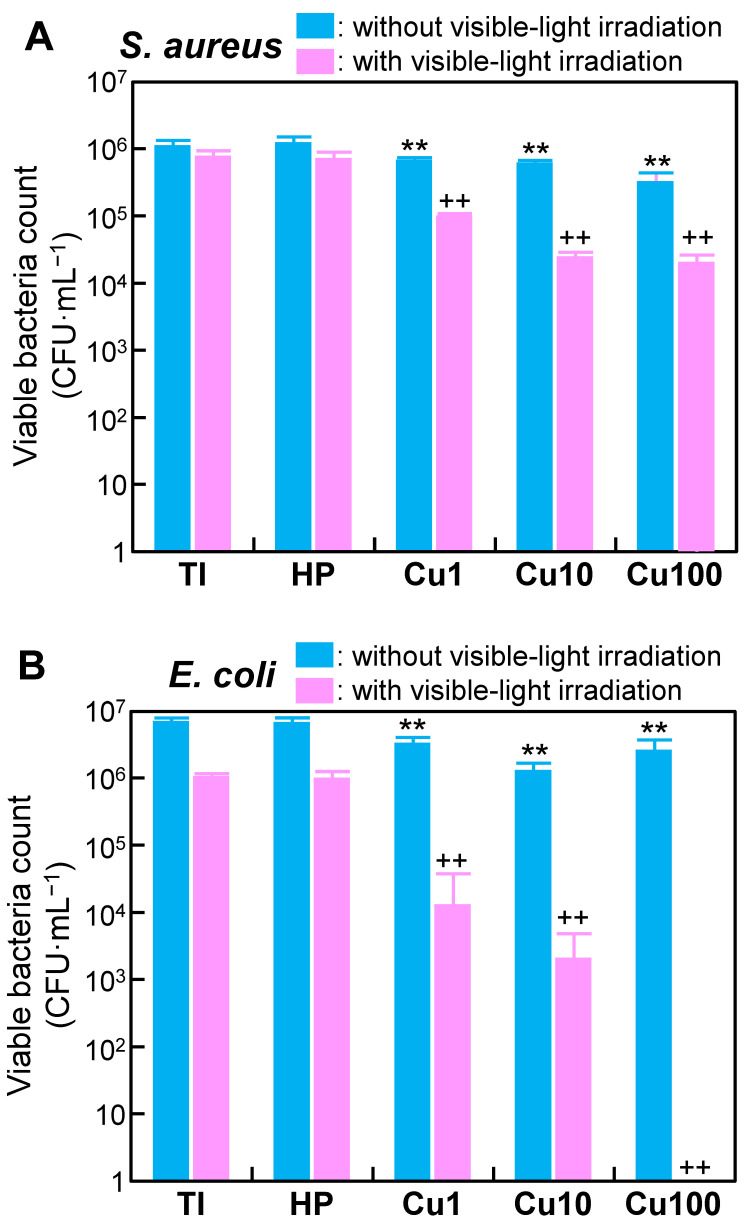
Viable counts of *S. aureus* (**A**) and *E. coli* (**B**) in samples without and with visible-light irradiation. Values are presented as arithmetic mean ± SD (*n* = 4). ** *p* < 0.01 vs. TI without visible-light irradiation, ++ *p* < 0.01 vs. TI with visible-light irradiation (one-way analysis of variance followed by multiple-hypothesis tests (Tukey’s honestly significant difference tests)). CFU: colony-forming unit, TI: sample with no treatment, HP: sample with H_2_O_2_ treatment, Cu1: sample with H_2_O_2_, 1 mM Cu(OAc)_2_, and heat treatment, Cu10: sample with H_2_O_2_, 10 mM Cu(Oac)_2_, and heat treatment, Cu100: sample with H_2_O_2_, 100 mM Cu(OAc)_2_, and heat treatment.

With or without visible-light irradiation, the viable counts of *S. aureus* and *E. coli* did not differ significantly when comparing between the HP and TI samples; in contrast, viable counts for both bacterial species were decreased significantly, with and without irradiation, in the Cu1, Cu10, and Cu100 samples (compared to the TI sample) (Figure 7). Notably, among samples irradiated with visible light, the viable counts of *E. coli* in the Cu1, Cu10, and Cu100 samples were decreased by more than two logs compared to similarly treated TI and HP samples.

**Figure 8:**
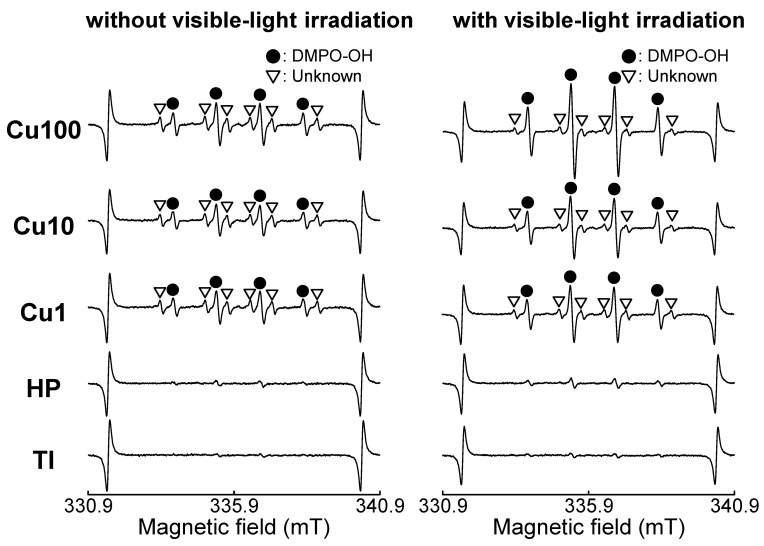
ESR spectra of samples without and with visible-light irradiation. ESR: electron spin resonance, DMPO: 5,5-dimethyl-1-pyrroline-*N*-oxide, TI: sample with no treatment, HP: sample with H_2_O_2_ treatment, Cu1: sample with H_2_O_2_, 1 mM Cu(OAc)_2_, and heat treatment, Cu10: sample with H_2_O_2_, 10 mM Cu(OAc)_2_, and heat treatment, Cu100: sample with H_2_O_2_, 100 mM Cu(OAc)_2_, and heat treatment.

The ESR spectra of the Cu1, Cu10, and Cu100 samples, with or without visible-light irradiation, exhibited two classes of ESR signals (Figure 8). The first showed hyperfine coupling constants of a_N_ = 1.49 and a_H_ = 1.49 mT and was assigned to DMPO-OH, a spin adduct of DMPO and ·OH; the second was an unknown signal. Notably, the intensity of the DMPO-OH peaks was increased by irradiation with visible light. The concentration of hydroxyl radicals (·OH) generated from the samples was elevated by irradiation with visible light, and the ·OH concentrations were nominally higher in the Cu1, Cu10, and Cu100 samples compared to the TI and HP samples (Table 3).

## 4. Discussion

Following sequential exposure to H_2_O_2_, Cu(OAc)_2_, and heat (the procedure described in the present work), the surface of the HP sample exhibited a fine network structure consisting of anatase- and rutile-type TiO_2_ (Figure 2 and Figure 3A). H_2_O_2_ has been reported to react with Ti to form TiO_2_ on Ti as follows [38].

Ti + 2H_2_O_2_ → TiOOH + 1/2O_2_ + H_3_O+ (or H^+^) (4)

TiOOH + 1/2O_2_ + H_3_O^+^ → Ti(OH)_4_ (5)

Ti(OH)_4_ → TiO_2_ + 2H_2_O (6)

The TiO_2_ fine network structure formed by immersion in H_2_O_2_ solution remained even after immersion in 100 mM Cu(OAc)_2_ solution, indicating that the undesired dissolution of the TiO_2_ layer seen upon exposure to strongly acidic Cu solutions was avoided by using a weakly acidic Cu(OAc)_2_ solution for Cu doping of the TiO_2_. We attribute the observed increase in the intensity ratio of the TiO_2_ XRD peaks (ratio of rutile-type to anatase-type) in the Cu1, Cu10, and Cu100 samples (Figure 2) to the enhancement of anatase-to-rutile transformation by Cu doping of the TiO_2_ [47,48,49,50,51].

Figure 2, Figure 3 and Figure 4 and Table 2 indicated that the small particles formed on the Cu1, Cu10, and Cu100 samples are composed primarily of CuO. There are concerns about the stability and durability of small CuO particles [52], but we believe that the small CuO particles are stable and chemically durable, given that the concentration of Cu released from samples was less than 0.8 ppm, even after soaking in PBS for 28 days (Figure 6), and that the color of samples remained black even after the soaking in PBS. Additionally, the data in Table 2 indicated that the Cu concentration on the sample surface does not increase linearly with the Cu(OAc)_2_ concentration, instead showing saturation at about 16 atomic%. In contrast, in our previous study on Ti subjected to NaOH-Cu(NO_3_)_2_-heat treatments [27], the Cu concentration on the sample surface peaked at approximately 12 atomic%. Although the mechanistic basis for this difference is unknown, the H_2_O_2_-Cu(OAc)_2_-heat treatment proposed in the present work permits the doping of nominally greater levels of Cu onto the sample surface than does the previous NaOH-Cu(NO_3_)_2_-heat treatments.

All samples other than the TI sample accumulated a dense and uniform surface apatite layer during 7 days of incubation in SBF (Figure 4). Previous work [33] has shown that the anatase- and rutile-type TiO_2_ on these sample surfaces (Figure 2) is responsible for the apatite formation during immersion in SBF. The apatite-forming ability of the Cu1, Cu10, and Cu100 samples appeared to be much higher than that seen previously in samples prepared by NaOH-Cu(NO_3_)_2_-heat treatments [28]. Therefore, the technique described here succeeded in forming Cu-doped TiO_2_ with increased apatite-forming ability on Ti. Many factors affect the apatite-forming ability of TiO_2_, including the acidity and basicity of Ti-OH groups [53,54,55], and the crystalline phase [33] and porosity [56] of TiO_2_. In addition, the apatite-forming ability of metal-doped TiO_2_ on Ti is affected by the chemical state of the doped metal [27]. To gain insight for the preparation of samples with higher apatite-forming ability, it is necessary to identify the factor(s) responsible for the higher apatite-forming ability of the present samples (compared to that of the previous samples). However, we are not able to define the relevant factor(s), given the many differences between the processes used in the present and previous work. Further study will be needed to identify the factor(s) responsible for the higher apatite-forming ability of the present samples. For clinical applications, samples prepared as in the present paper will need to be evaluated in vivo for their bone-binding ability. We conjecture that samples prepared as described here will form an apatite layer on their surface even in vivo, and that this apatite layer will facilitate direct bonding to living bone [42].

The Cu1, Cu10, and Cu100 samples also showed antibacterial activity against *S. aureus* and *E. coli*, even in the absence of visible-light irradiation (Figure 7). This activity may reflect the combined effects of copper ions released from the sample (Figure 6), contact between bacteria and small CuO particles present on the sample surface [57], and the small amount of ·OH generated from the samples (Table 3). The antibacterial activity of these samples was remarkably enhanced by visible-light irradiation. The generation of ·OH from these samples (Figure 8 and Table 3) may be responsible for the enhanced antibacterial activity of these samples following irradiation with visible light. Notably, the improvement in the antibacterial activity following visible-light irradiation appeared to be more pronounced against *E. coli* than against *S. aureus* (Figure 7B). This distinction may reflect the fact that the peptidoglycan layer in *E. coli*, a Gram-negative bacterium, is thinner than that that in *S. aureus*, a Gram-positive bacterium [58,59]. Given that ROS, including ·OH, can induce chronic inflammation, future in vivo studies will be needed to investigate any inflammatory responses induced by ROS generated by the samples. However, we conjecture that the inflammatory response(s) induced by ROS generated by the samples will be limited, since the concentration of ·OH was low, and increased only when the sample was irradiated with visible light (Table 3). The antibacterial activity of the samples against other bacteria that cause peri-implantitis, such as *Porphyromonas gingivalis*, should also be investigated in future studies.

In the ESR assessment, DMPO-OH peaks were observed for the Cu1, Cu10, and Cu100 samples, even in the absence of visible-light irradiation (Figure 8). This observation indicated that these samples generate ·OH even in the dark (Table 3). We hypothesize that ·OH is generated when CuO particles present on the sample surface react with the reagent or water used in this experiment [60,61]. The unknown peaks in the ESR spectrum of the sample may be attributable to carbon-centered radicals, such as methyl radicals [62]. These carbon-centered radicals may originate from carbon in reagents decomposed by CuO particles present on the sample surface.

To the eye, the Cu1, Cu10, and Cu100 samples appeared black in color (inset photographs in Figure 3A). This black coloration presumably is attributable to the visible light absorption of the CuO [31,63,64,65,66,67,68] formed on these samples by heat treatment. For CuO-doped TiO_2_, it has been reported that CuO doping extends the tailing of the absorption edge toward the visible region, resulting in band-gap narrowing [64,65,66]. In addition, the precipitation of fine particles of CuO on the TiO_2_ surface (Figure 2) suggests that the visible-light-responsive photocatalytic effect may have been induced by interfacial charge transfer of electrons from TiO_2_ to CuO [31,67,68]. Although no clear UV-visible spectrum (which would indicate narrowing of the band gap) was obtained for these samples, we speculate that the Cu-doped TiO_2_ formed on Ti by this method exhibits visible-light-responsive photocatalytic activity due to the interface charge transfer between CuO and TiO_2_; this activity would result in the observed enhancement of antibacterial activity by visible-light irradiation (Figure 7). Additionally, as shown in Figure 2, CuO small particles formed sparsely on Ti substrate, making it difficult to obtain clear UV-visible spectra of the CuO small particles themselves.

In the present study, we found that H_2_O_2_-Cu(OAc)_2_-heat treatments induced the layering of TiO_2_ with CuO fine particles; we further demonstrated that the TiO_2_ layer showed apatite-forming ability during incubation in SBF and visible-light-enhanced antibacterial activity. The novelty of our work is that we have succeeded in preparing titanium substrates with CuO-supported TiO_2_ surfaces; these substrates exhibited excellent apatite-forming ability and visible-light-enhanced antibacterial activity against both *E. coli* and *S. aureus*. Previous work has shown that apatite-coated TiO_2_ powders show antibacterial activity against *E. coli* and *S. aureus* when irradiated with black light or visible light [69], and that TiO_2_ layers formed on Ti substrates by Au-sputtering and thermal oxidation show antibacterial activity against *E. coli* when irradiated with visible light [70]. However, to the best of our knowledge, surface-modified titanium substrates with similar functions have not been reported to date. Future investigations will be needed to evaluate the toxicity and bone-bonding ability of samples prepared as described here. Nonetheless, we hypothesize that dental implants with Cu-doped TiO_2_ on the abutments may decrease the risk of peri-implantitis when treated in the dental clinic by regular or on-demand irradiation with visible light. Furthermore, if employed for devices that are partially exposed outside the body (such as external fixators), materials with such surfaces are expected to exhibit antibacterial properties when exposed to visible light.

## 5. Conclusions

A network structure of anatase- and rutile-type TiO_2_ with CuO fine particles was generated when Ti was subjected to H_2_O_2_-Cu(OAc)_2_-heat treatments. Such surface-treated Ti accumulated a dense and uniform surface layer of apatite when incubated for 7 days in SBF, indicating enhanced apatite-forming ability compared to that previously reported for Ti subjected to NaOH-Cu(NO_3_)_2_-heat treatments. Furthermore, samples prepared as described here showed enhanced antibacterial activity against *E*. *coli* and *S. aureus* when subjected to visible-light irradiation. The increased antibacterial activity of these samples under visible-light irradiation may be attributable to the generation of ·OH by the samples. Together, these results suggest that Ti implants with reduced risk of peri-implantitis following on-demand visible-light irradiation can be obtained by the H_2_O_2_-Cu(OAc)_2_-heat treatments described here.

## Figures and Tables

**Table 1:** Surface treatments and names of samples.

Surface Treatment	Sample Name
None	TI
H_2_O_2_ + heat	HP
H_2_O_2_ + 1 mM Cu(OAc)_2_ + heat	Cu1
H_2_O_2_ + 10 mM Cu(OAc)_2_ + heat	Cu10
H_2_O_2_ + 100 mM Cu(OAc)_2_ + heat	Cu100

**Table 2:** Concentrations of Cu and proportions of Cu species at the surfaces of the Cu1, Cu10, and Cu100 samples. at.%: atomic percent, Cu1: sample with H_2_O_2_, 1 mM Cu(OAc)_2_, and heat treatment, Cu10: sample with H_2_O_2_, 10 mM Cu(OAc)_2_, and heat treatment, Cu100: sample with H_2_O_2_, 100 mM Cu(OAc)_2_, and heat treatment.

Sample	Concentration of Cu (at.%)	Proportion of Cu Species (%)	
		Cu^0^ + Cu^+^	Cu^2+^
Cu1	6.8	20.1	79.9
Cu10	15.8	19.2	80.8
Cu100	12.9	26.5	73.5

**Table 3:** Concentrations of hydroxyl radical (·OH) generated by samples with and without visible-light irradiation. TI: sample with no treatment, HP: sample with H_2_O_2_ treatment, Cu1: sample with H_2_O_2_, 1 mM Cu(OAc)_2_, and heat treatment, Cu10: sample with H_2_O_2_, 10 mM Cu(OAc)_2_, and heat treatment, Cu100: sample with H_2_O_2_, 100 mM Cu(OAc)_2_, and heat treatment.

Sample	·OH Concentration (µM)	
	Without Visible-Light Irradiation	With Visible-Light Irradiation
TI	0.09	0.19
HP	0.21	0.51
Cu1	1.08	3.03
Cu10	1.44	3.23
Cu100	1.50	4.36

## Data Availability

The raw data supporting the conclusions of this article will be made available by the authors on request.
